# Earth's core–mantle boundary shaped by the crystallization of a hydrous terrestrial magma ocean

**DOI:** 10.1093/nsr/nwae169

**Published:** 2024-05-13

**Authors:** Qingyang Hu, Jie Deng, Yukai Zhuang, Zhenzhong Yang, Rong Huang

**Affiliations:** Center for High Pressure Science and Technology Advanced Research (HPSTAR), China; Shanghai Advanced Research in Physical Sciences (SHARPS), China; Department of Geosciences, Princeton University, USA; Institute of Atomic and Molecular Physics, Sichuan University, China; Key Laboratory of Polar Materials and Devices, Department of Electronics, East China Normal University, China; Key Laboratory of Polar Materials and Devices, Department of Electronics, East China Normal University, China

## Abstract

Enigmatic anomalous structures of Earth's lowermost mantle may have been incubated at the hydrous magma ocean of the Hadean eon.

A water-rich global magma ocean (MO) likely existed in the early Hadean eon and subsequently cooled and solidified. While water is an oxidant and is known to fundamentally affect liquid crystallization, the role of primordial water during the MO crystallization stage has rarely been considered. Recent breakthroughs in water-mineral chemistry suggest that the solidification of a hydrous MO may favor the formation of pyrite-type (Py-) (Mg_1-_*_x_*, Fe*_x_*)O_2_. Experiments reveal that this Py-phase has the strongest affinity for ferrous Fe among the major deep mantle phases. This perspective will focus on the role of Py-phase in creating the ancient structures of Earth's core–mantle boundary (CMB). Many subsequent processes that have shaped the mantle and core may be relevant to this primordial water chemistry and have been incubated since the terrestrial MO of the Hadean eon.

A Moon-forming giant impact likely extensively melted the early Earth, leading to the formation of a global MO and setting the initial conditions for Earth's mineralogy, geodynamics and evolution. The MO quickly cooled and solidified, marking the most drastic chemical differentiation events of the silicate Earth. The detailed processes of the giant impact are still under debate (Supplementary Data), but it is generally agreed that the solidification and differentiation of an MO are dynamic, with chemical compositions of residual melts and crystallized solid phases evolving along with sedimentation of mineral precipitates. According to the partitioning behaviors of major elements, the residual melts become increasingly FeO-rich and SiO_2_ depleted with crystallization [1,2]. These dense Fe-rich melts form resides at the bottom of the mantle, forming the basal MO (BMO). The formation of the BMO may be inevitable, which is supported by thermal evolution models, high-pressure experiments, *ab initio* calculations and geodynamic modeling [1] (ongoing issues of the BMO are discussed in the Supplementary Data). The BMO at the late stage is thus, likely, much less silicic than pyrolite, and the first phase to crystalize under a dry (water-free) condition shifts to ferropericlase (Fp), instead of bridgmanite [2].

We note, however, that this picture of MO crystallization is only valid when the MO is dry. To our best knowledge, the solidification of the MO under a wet condition has rarely been explored. In fact, the MO following the giant impact has been suggested to host a substantial amount of volatiles like water [3]. Water may profoundly change the liquidus phase [4]. As H is highly incompatible, the BMO is not only enriched in Fe but also in water, and becomes more hydrous with crystallization. Subsequently, water oxidizes Fp to the Py-type H-bearing (Mg_1-_*_x_*, Fe*_x_*)O_2_H*_y_* (0 ≤ *x* ≤ 1, 0 ≤ *y* ≤ 1) in Earth's lower mantle [5] (Reaction 1), and reacts with Fe at the CMB [6] (Reaction 2).


(Reaction 1) \begin{eqnarray*}
&&\left( {{\mathrm{M}}{{{\mathrm{g}}}_{1 - x}},{\mathrm{F}}{{{\mathrm{e}}}_x}} \right)\!{\mathrm{O}} + {{{\mathrm{H}}}_2}{\mathrm{O}}\\
&& \to \left( {{\mathrm{M}}{{{\mathrm{g}}}_{1 - x}},{\mathrm{F}}{{{\mathrm{e}}}_x}} \right)\!{{{\mathrm{O}}}_2}{{{\mathrm{H}}}_y}\left( {{\mathrm{Py}}} \right) + {{{\mathrm{H}}}_2}\\
\end{eqnarray*}

(Reaction 2) \begin{eqnarray*}
{\mathrm{Fe}} + {{{\mathrm{H}}}_2}{\mathrm{O}} \to {\mathrm{Fe}}{{{\mathrm{O}}}_2}{{{\mathrm{H}}}_x}\left( {{\mathrm{Py}}} \right) + {\mathrm{Fe}}{{{\mathrm{H}}}_y}\end{eqnarray*}

Obviously, the fraction and stability of Py-phase hinges on the availability of water, its density and Fe content. There is an urgent need to understand the partitioning of Fe-Mg among Py-(Mg_1-_*_x_*, Fe*_x_*)O_2_ and other deep mantle phases.

Fe-Mg partitioning is well established in typical dry lower mantle mineral systems and Fp is more favorable to ferrous Fe than other major constituents of the lower mantle [1]. However, when water is involved and the extra Py-phase presents, such partitioning and the resulting Fe distribution among mantle phases remain unknown. This key information is obtained by quenching Fp and Py-(Mg, Fe)O_2_ assemblages from high-pressure conditions. Previous experiments have successfully synthesized the Py-phase from water and Fp with 60∼80 mol% of MgO, which is a reasonable approximation of the composition of Fp in Earth's lower mantle. Here, we synthesized a sample from Fp containing 60 mol% MgO and examined the Fe-Mg partitioning under wet conditions. The starting materials were heated at 84 GPa and 2300 K under water saturation. After decompression to ambient conditions, the Fe-Mg ratio on the sample surface remained almost unchanged [5]. However, we noticed textures of compositional variation by lifting out a perpendicular lamella of the sample using a focused ion beam (Figs S1 and S2). Under high-resolution transmission electron microscopy (HR-TEM) imaging, the thinned lamella features brighter bands with a higher Fe concentration. The texture of such bands is amorphous (Fig. S3), suggestive of sluggish crystallization. Scanned TEM energy-dispersive X-ray spectroscopy mapping shows that the Fe-Mg ratio *X*_Fe_/(*X*_Mg_ + *X*_Fe_) from regions of interest is 0.84(4) (Table S1), indicating strong Fe-Mg partitioning in band regions. Within the bands, the cation to anion ratio (*X*_Mg_ + *X*_Fe_)/*X*_O_ equals 0.47(6), which is in agreement with the excessive O amount of Py-phase and confirms their origins from the depressurization of the Py-(Mg_0.16_, Fe_0.84_)O_2_. The resulting exchange coefficient *K_D_*is 0.131(12). Py-phase's strong affinity to Fe is verified by similar experiments on Fp with 20∼30% of Fe at 95–121 GPa, in which nearly complete decomposition of Py-phase to FeO_2_ was observed [7].

The drastic Fe-Mg partitioning directly affects the structure of the CMB. The global MO created by the Moon-forming giant impact contains an appreciable amount of volatiles, among which water is fully miscible in silicate melts at pressures beyond ∼5 GPa [3] and concentrates in residual melts as MO solidification proceeds [1] (Fig. 1). Crystallizing hydrous SiO_2_-poor and FeO-enriched melts leads to the formation of Py-phase [5]. While it is generally accepted that the BMO becomes increasingly FeO-rich, the strong Fe-Mg partition causes the stabilized Py-phase to drain ferrous Fe out of the BMO. As a result, when the bulk composition of the system becomes progressively FeO-rich and SiO_2_-poor, the first phase to crystalize will shift from bridgmanite to Fp in a dry condition and to Py-phase in a wet condition. The mineral composition of the CMB is then refreshed as the Fe-poor post-perovskite/perovskite phase, Fe-dominant Py-(Fe, Mg)O_2_ and Fe-poor Fp.

**Figure 1.**
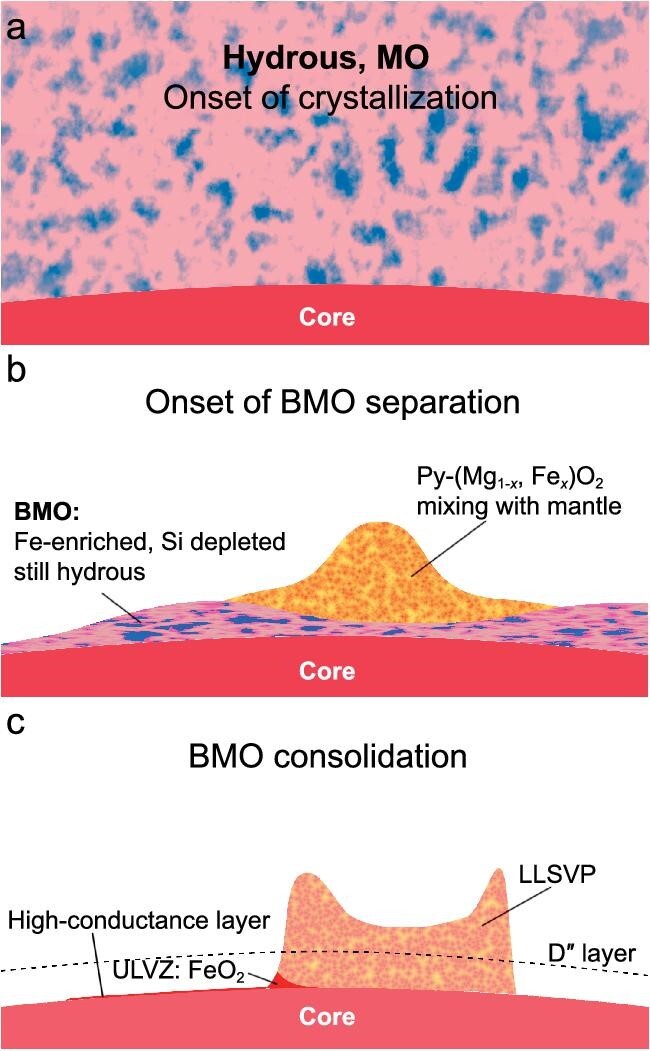
Schematic plot for hydrous BMO crystallization. (a) Onset of hydrous MO crystallization. Blue indicates that the MO is water-enriched. (b) The formation of the BMO with a higher concentration of Fe and water. Thermochemical piles made of Py-(Mg_1-_*_x_*, Fe*_x_*)O_2_ are pushed up above the BMO and those piles will mix with major mantle components. (c) After the BMO solidifies, nearly all Fe will partition into the Py-phase at the lowermost mantle. Those Py-FeO_2_-rich regions may form heterogeneous structures like the high-conductance layer [8] and the ULVZs [9,11] above the CMB.

We then estimate the amount of Py-phase at the CMB by assuming a conservative lower bound of 1.0–4.0 ocean masses as the water budget, and propagating numbers through the partition of water into melts and its retention during crystallization. Our calculations suggest that the water retained in the BMO may generate Py-FeO_2_ that amounts to an approximately 1.1–4.2 km shell embracing the CMB (calculation details in the online Supplementary Data) in Py-phase solid solutions. The electric conductivity of Py-FeO_2_ is at the level of 10^4.5^ S/m and is further boosted by the superionic conduit [6]. A kilometer-thick layer of Fe-enriched Py-phase alone may approach the required high conductance (∼10^8^ S) that explains the observed electromagnetic coupling between the fluid outer core and mantle. If there is regional heterogeneity of water in the BMO, the abundance of Py-phase would vary accordingly. For example, the high conductance in the lowermost mantle under the Pacific [8] may indicate the mesoscale formation of a Py-FeO-rich thin layer.

The cumulation of Py-phase likely mixes with the ambient mantle and the resulting structures are readily detectable through seismology. A mixture with 40%–50% of Py-FeO_2_ and the ambient mantle can well fit the seismic features of ultra-low velocity zones (ULVZs) (Fig. 1c) [9]. We emphasize that the amount of Py-phase derived here is based on the lower-bound water content of MO. For a more realistic water content, Py-phase may even engage in forming larger low-seismic structures by mixing with the lower mantle materials through thermal convection. The other important product of the primordial water reaction, hydrogen, has been proposed to be more siderophile than oxygen at conditions of core formation [10]. That means a substantial amount of H may infiltrate into the liquid outer core, oxidizing the mantle side. The Py-phase will only be more preferable in such an oxidized environment and the residual H in silicates/oxides will continue to diffuse as super-ions, creating a chemically heterogenous CMB.

In short, we propose that the CMB heterogeneities may be incubated in a hydrous MO. The solidification of a hydrous MO produces an exotic oxidized Py-phase, chemically distinct from the mantle materials. Its strong affinity to ferrous Fe dynamically stabilizes it at the CMB, forming patches featuring low-seismic velocities and high electrical conductivity. Our results suggest that the lowermost low-seismic-velocity patches may originate from Fe-rich Py-phase solidified from a wet MO. This Fe preference also results in a stark density contrast between Py-phase-enriched patches and the surrounding mantle, making such heterogenous structures dynamically stable for a prolonged period of time. This model suggests that the lowermost mantle heterogeneity may be long-lived, aligning with recent numerical modeling results [11].

## Supplementary Material

nwae169_Supplemental_File
