# Supplementary material for: Earth's core–mantle boundary shaped by the crystallization of a hydrous terrestrial magma ocean
Source: Natl Sci Rev. 2024 May 13;11(7):nwae169. doi: 10.1093/nsr/nwae169 (PMC11173207; doi:10.1093/nsr/nwae169)
Supplement: nwae169_Supplemental_File [file nwae169_supplemental_file.pdf]

SUPPLEMENTARY MATERIALS

Assumptions for a hydrous terrestrial magma ocean

The chemical reactions of primordial water hinge on the formation of a basal magma ocean (BMO) where both water and FeO are progressively enriched. Although many previous studies suggest that a BMO may be an inevitable consequence of the giant impact at the early history of Earth [1-6], details of this giant impact are still under debate. For example, whether it belongs to a high-energy (melt the entire mantle) or low-energy (partially melt the mantle) event [2]. If it is a high-energy event, BMO may be formed by the separation of mantle originated from the density crossover of silicate melts and solids [3, 7, 8]. On the other hand, a low-energy impact leads to the formation of a BMO if the remanent of impacted planet, the Theia, remains partially molten [1]. Regardless of the nature of giant impact, this study assumes that a BMO existed in the early Earth given the growing number of evidence supporting the formation of BMO [4, 6].

A BMO, by definition, should be FeO-rich to be gravitationally stable at the base of the mantle. Here, we discuss whether a BMO stores sufficient water to trigger the Reactions 1 and 2 in the main text. The source of water is an outstanding question, with many mechanisms proposed in the literature, such as water formed by the chemical reaction of nebula gas and MOs [9] and direct delivery by late veneer [10]. Those mechanisms lead to quite different redox conditions and various initial water inventory. Nevertheless, we emphasize that that this study makes reasonable estimation of water budget and the water will accumulate in the BMO. Specifically, our work suggests that 1.0-4.0 oceans of water are sufficient to exhibit visible anomalies at the core-mantle boundary (CMB). Either the source of water was endogenic [9, 11, 12] or exogenic [10, 13], this range aligns with the lower bound estimates of water inventory for the bulk mantle of today [14]. An equally important issue is the retention of water. In fact, both numerical modelling and mineral physics experiments have implied that much of water would be stored in the mantle during crystallization [15]. For example, the assumption of large water retention is supported by Nakajima and Stevenson [16], who computed the thermal structure of the Moon-forming disk and suggested that the amount of lost H_2O/H_2 would have been small. Miyazaki and Korenaga [17] are more conservative on this point and they think Earth’s volatile concentrations are close to the threshold of degassing one ocean water. On the other hand, mineral physics experiments show that water strongly concentrates in the melts [18], making the residual melts progressively water-rich with crystallization. A corollary is that the BMO can be highly enriched in water in the late stage of crystallization even if it starts with a low initial amount of H_2O [19]. Previous works have predicted a BMO may end up with up to 2 wt.% of water [20], or 7.0 masses of ocean water [21], which are way above the amount needed for our proposed

CMB structures. In comparison, our estimations of 1.0-4.0 oceans of water budget are *de facto* more conservative for proposed reactions.

Transmission electron microscopy

The high-pressure sample was initially picked out from the gasket chamber. We used FIB to lift out a sample cross-section and polished the lamella until the thickness was less than 100 nm. After that, the lamella is ready for TEM and composition investigation. The microstructure images of the quenched sample were obtained using a JEOL-2100F field-emission TEM equipped with an energy dispersive X-ray detector and operated at 200 kV during the experiment.

Estimation of Py-phase at the CMB

This section itemizes our calculation for the abundance of Py-phase formed during the hydrous MO crystallization and its uncertainties. Our calculation starts with 1.0-4.0 ocean mass of water budget (one ocean mass is 1.347×10^{21} kg) [22, 23]. It is well-known that water strongly partitions into silicate melts [24, 25]. On the other hand, outgassing of H is minimal at the stage of BMO and shallow MO separation, *e.g.* less than 1% depletion for 1.0 ocean H budget [15]. Therefore, the BMO has stored a substantial amount of primordial water.

At 50% crystallization of MO, we employed the exchange coefficient of water in melt over silicate as $\log_{10}D_{water} = -1.0$ which should be the upper limit to our best knowledge. Here, D_{water} equals to the amount of water in silicate over that in melts. The minimum amount of water in BMO is then calculated as $1.225\text{-}4.900\times10^{21}$ kg. We made three assumptions for the amount of Py-FeO_2 cumulated at the lowermost mantle. In the first place, we employ the outgassing of H from previous numerical model, in which H depletion proportion is minimal for 1.0 ocean water budget and ~5.0% for 4.0 ocean water budget. These values are valid for both equilibrium and fractional crystallization at 40% melt and above [15].

Secondly, we hypothesize that water in the BMO reacts completely to form the Py-phase. Since the Py-phase should be iron enriched, our last assumption thinks Py-phase will sink to the lowermost mantle, where the Mg-Fe exchange coefficient approaches zero [26]. The mass of Py-FeO_2 is calculated by:

$$\frac{m_{water}}{M_{\mathrm{H_2O}}} = \frac{m_{\mathrm{FeO_2}}}{M_{\mathrm{FeO_2}}} \qquad (13)$$

where M_{H_2O} and M_{FeO_2} are the atomic mass of H_2O and FeO_2, m_{water} is 1.225-4.9000×10^{21} kg. Experiment has suggested K_D close to zero at the CMB conditions, we therefore reasonably assume all Fe partitions into the Py-phase [26]. This Fe-Mg partition can be further quantified through theoretical calculation and high-pressure experiments. The mass of FeO_2 is then calculated as 5.853×10^{21} to 2.224×10^{22} kg (account 5% H depletion). Given by the established equation of state data [27] with P-T conditions of 130 GPa and 3,000 K, the volume of Py-FeO_2 can be derived as V_{Py} = 1.66-6.31×10^{17} m^3. We use r_{CMB} = 3477 km as the radius of CMB. The thickness of Py-phase layer equals to $V_{Py}/(4\pi r_{CMB}^2)$ = 1.1-4.2 km. The uncertainty of thickness is within 5%, propagated from the equation of state and r_{CMB} data.

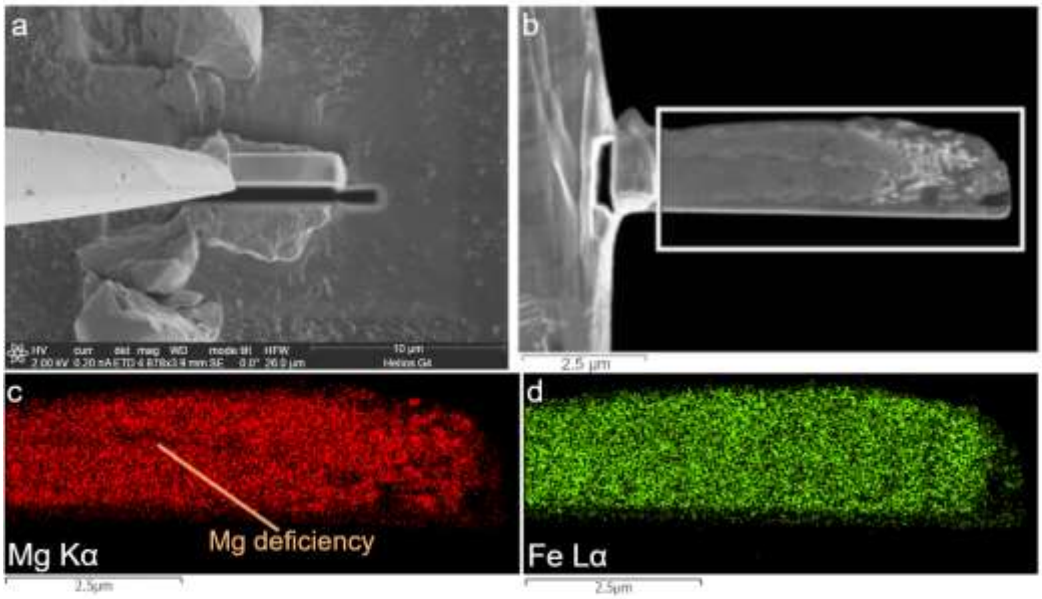

Supplementary Materials Fig. S1. Preparing a TEM sample. a, After transferring the sample from diamond anvil cell chamber on a carbon tape, we fist cut a cross section with size dimensions of 8μm (length) ×2μm (width) ×2μm (thickness). **b,** Sample after FIB thinning. **c-d,** Rough EDS mapping for the distribution of Mg (**c**) and Fe (**d**). Magnesium deficiency is detected at the center part of the sample.

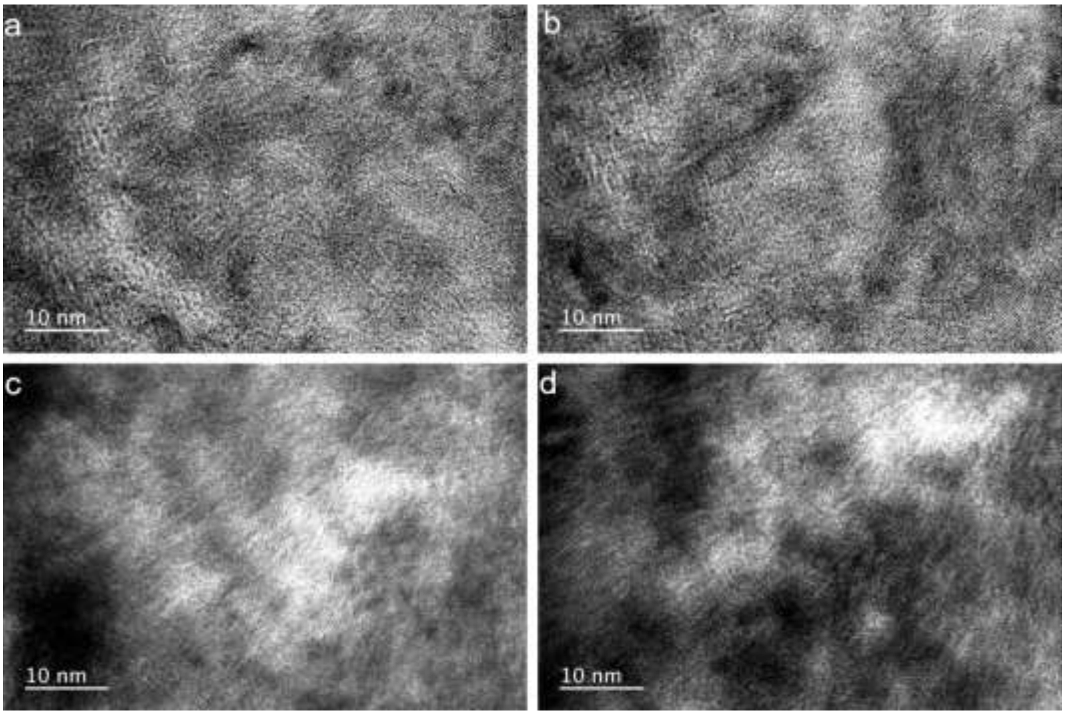

Supplementary Materials Fig. S2. Morphology of the lamella for TEM experiment. a-b, HR-TEM images In the Mg enriched regions. The sample crystallized into small but isolated grains. **c-d,** HR-TEM in the brighter and more Fe enriched region. We observed radial topography along the edge, suggesting amorphous structure.

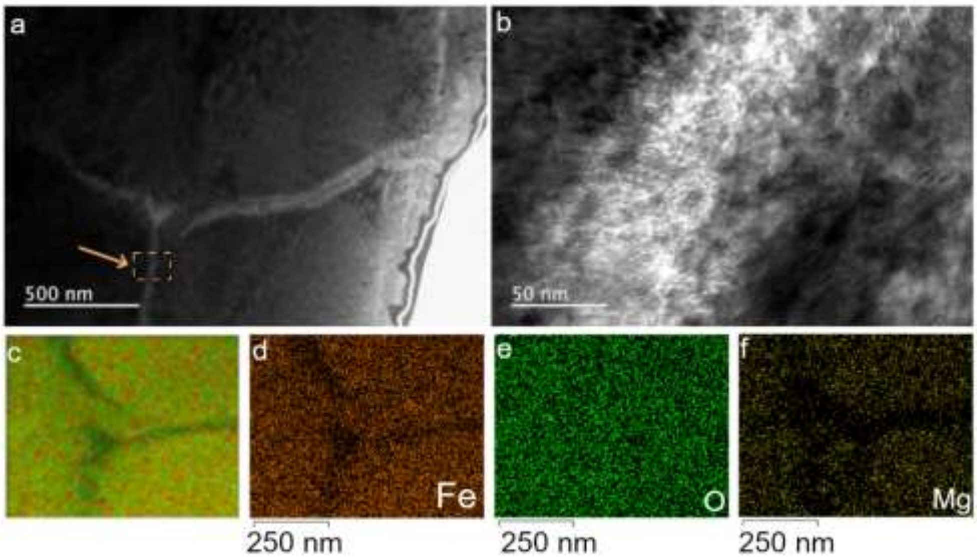

Supplementary Materials Fig. S3, Mg-Fe distribution in a cross-section of sample. a, a TEM image of the FIB thinned sample. **b,** HR-TEM image corresponding to the square region in **a. c,** Annular dark-field (ADF) image of the interested area for EDS mapping. **d-f,** EDS mapping of Fe, O and Mg over the area of interest subfigure **c**, respectively.

Supplementary Materials Table S1. Compositional analysis of the quenched sample in TEM-EDS.

Element	Orbital	*k* factor	Absorption adjustment	wt%	wt% sigma	Atomic ratio
O	K-edge	1.116	1.07	39.94	1.17	67.80
Mg	K-edge	0.592	1.11	4.73	0.45	5.29
Fe	L-edge	0.607	0.98	55.33	1.15	26.91
Total				100.0		100.0

REFERENCES

1. Yuan Q, Li M, Desch SJ *et al*. Moon-forming impactor as a source of Earth's basal mantle anomalies. *Nature* 2023; **623**:95-9.
2. Schaefer L and Elkins-Tanton LT. Magma oceans as a critical stage in the tectonic development of rocky planets. *Philos Trans A Math Phys Eng Sci* 2018; **376**.
3. Caracas R, Hirose K, Nomura R *et al*. Melt–crystal density crossover in a deep magma ocean. *Earth Planet Sci Lett* 2019; **516**:202-11.
4. Labrosse S, Hernlund J and Hirose K. Fractional melting and freezing in the deep mantle and implications for the formation of a basal magma ocean. In: James Badro & Walter M (eds.). *The Early Earth: Accretion and Differentiation*: American Geophysical Union; 2015. 123-42.
5. Labrosse S, Hernlund JW and Coltice N. A crystallizing dense magma ocean at the base of the Earth's mantle. *Nature* 2007; **450**:866-9.
6. Boukaré CE, Ricard Y and Fiquet G. Thermodynamics of the MgO-FeO-SiO_2 system up to 140 GPa: Application to the crystallization of Earth's magma ocean. *J Geophys Res: Solid Earth* 2015; **120**:6085-101.
7. Andrault D. Thermodynamical constraints on the crystallization of a deep magma-ocean on Earth. *C R Geosci* 2019; **351**:221-8.
8. Andrault D, Bolfan-Casanova N, Bouhifd MA *et al*. Toward a coherent model for the melting behavior of the deep Earth's mantle. *Phys Earth Planet Inter* 2017; **265**:67-81.
9. Young ED, Shahar A and Schlichting HE. Earth shaped by primordial H_2 atmospheres. *Nature* 2023; **616**:306-11.
10. Drake MJ. Origin of water in the terrestrial planets. *Meteorit Planet Sci* 2004; **39**:A31.
11. Ikoma M and Genda H. Constraints on the mass of a habitable planet with water of nebular origin. *Astrophys J* 2006; **648**:696.
12. Kite ES and Schaefer L. Water on Hot Rocky Exoplanets. *Astrophys J Lett* 2021; **909**:L22.
13. Izidoro A and Piani L. Origin of water in the terrestrial planets: Insights from meteorite data and planet formation models. *Elements* 2022; **18**:181-6.
14. Ohtani E. Hydration and dehydration in Earth's interior. *Annu Rev Earth Planet Sci* 2021; **49**:253-78.
15. Bower D, Hakim K, Sossi P *et al*. Retention of water in terrestrial magma oceans and carbon-rich early atmospheres. *Planet Sci J* 2022; **3**:93.

16. Nakajima M and Stevenson DJ. Inefficient volatile loss from the Moon-forming disk: Reconciling the giant impact hypothesis and a wet Moon. *Earth Planet Sci Lett* 2018; **487**:117-26.
17. Miyazaki Y and Korenaga J. Inefficient water degassing inhibits ocean formation on rocky panets: An insight from self-consistent mantle degassing models. *Astrobio* 2022; **22**:713-34.
18. Hauri EH, Gaetani GA and Green TH. Partitioning of water during melting of the Earth's upper mantle at H_2O-undersaturated conditions. *Earth Planet Sci Lett* 2006; **248**:715-34.
19. Xie L, Walter M, Katsura T *et al*. Crystallization of a hydrous magma ocean in the shallow lower mantle. *Earth Planet Sci Lett* 2024; **633**:118651.
20. Du Z, Deng J, Miyazaki Y *et al*. Fate of hydrous Fe-rich silicate melt in Earth's deep mantle. *Geophys Res Lett* 2019; **46**:9466-73.
21. Wu Z, Song J, Zhao G *et al*. Water-induced mantle overturns leading to the origins of Archean continents and subcontinental lithospheric mantle. *Geophys Res Lett* 2023; **50**:e2023GL105178.
22. Abe Y and Matsui T. Early evolution of the Earth: Accretion, atmosphere formation, and thermal history. *J Geophys Res: Solid Earth* 1986; **91**:E291-E302.
23. Marty B. The origins and concentrations of water, carbon, nitrogen and noble gases on Earth. *Earth Planet Sci Lett* 2012; **313-314**:56-66.
24. Murakami M, Hirose K, Yurimoto H *et al*. Water in Earth's lower mantle. *Science* 2002; **295**:1885-7.
25. Ishii T, Ohtani E and Shatskiy A. Aluminum and hydrogen partitioning between bridgmanite and high-pressure hydrous phases: Implications for water storage in the lower mantle. *Earth Planet Sci Lett* 2022; **583**:117441.
26. Yang Z, Yuan H, Liu L *et al*. Chemical reaction between ferropericlase (Mg,Fe)O and water under high pressure-temperature conditions of the deep lower mantle. *Am Mineral* 2023; **108**:530-5.
27. Liu J, Hu Q, Kim DY *et al*. Hydrogen-bearing iron peroxide and the origin of ultralow-velocity zones. *Nature* 2017; **551**:494.
